# An Experimental Investigation of Lipovaccines, with a Note on Triple Dysentery Lipovaccines

**Published:** 1918-05

**Authors:** 


***An Experimental Investigation of Lipovaccines, with a Note on Triple Dysentery Lipovaccines.*** By E. R. Whitmore, Lieut.-Col. M. C., U. S. Army, and E. A. Fennel (Cincinnati), Lieut. M. C., U. S. Army. *Journ. A. M. A.*, March 30, 1918.

The authors found that the lipovaccines (Le Moignic) can be made on a large scale by growing the bacteria in Kolle flasks; taking off the growth with a vacuum scraper; freezing and drying in vacuo, and emulsifying in lanolin and oil by grinding in a ball mill, using glass bottles and steel balls.

The oils can be sterilized by steam at 15 pounds for 15 minutes; by heating to 90 C. for 10 hours on a water bath, or by mixing with potassium iodide.

A dose of 3,000 million Shiga, 3,200 million " Y ", and 2,200 million Flexner type of the dysentery bacillus in oil may be given to men without marked local or general reaction. There appears to be production of agglutinins, precipitins, and bacteriolysins in the blood of vaccinated animals and men, and some evidence of complement fixation.

